# Automatic Non-Destructive Growth Measurement of Leafy Vegetables Based on Kinect

**DOI:** 10.3390/s18030806

**Published:** 2018-03-07

**Authors:** Yang Hu, Le Wang, Lirong Xiang, Qian Wu, Huanyu Jiang

**Affiliations:** 1 College of Biosystems Engineering and Food Science, Zhejiang University, 866 Yuhangtang Road, Hangzhou 310058, China; yhu@zju.edu.cn (Y.H.); wangle5994@zju.edu.cn (L.W.); lrxiang@zju.edu.cn (L.X.); wuqianhz@zju.edu.cn (Q.W.); 2 Key Laboratory of On Site Processing Equipment for Agricultural Products, Ministry of Agriculture, Beijing 100125, China

**Keywords:** plant growth measurement, Kinect v2, non-destructive, point cloud, 3D reconstruction

## Abstract

Non-destructive plant growth measurement is essential for plant growth and health research. As a 3D sensor, Kinect v2 has huge potentials in agriculture applications, benefited from its low price and strong robustness. The paper proposes a Kinect-based automatic system for non-destructive growth measurement of leafy vegetables. The system used a turntable to acquire multi-view point clouds of the measured plant. Then a series of suitable algorithms were applied to obtain a fine 3D reconstruction for the plant, while measuring the key growth parameters including relative/absolute height, total/projected leaf area and volume. In experiment, 63 pots of lettuce in different growth stages were measured. The result shows that the Kinect-measured height and projected area have fine linear relationship with reference measurements. While the measured total area and volume both follow power law distributions with reference data. All these data have shown good fitting goodness (*R*^2^ = 0.9457–0.9914). In the study of biomass correlations, the Kinect-measured volume was found to have a good power law relationship (*R*^2^ = 0.9281) with fresh weight. In addition, the system practicality was validated by performance and robustness analysis.

## 1. Introduction

Non-destructive plant growth measurement is the basis for many fields which focus on plant growth and health research, including phenotyping and breeding, crop production forecast, fertilizer and water management and other related applications. Height, leaf area, volume and biomass [1] are the major growth parameters of commonly researched plants, especially for leafy vegetables. Manual measurement for these parameters can be time-consuming and labor-intensive, thus the study of automatic and non-destructive measurement technology is important and necessary. For many years, a lot of studies have been carried out focusing on this issue, with different devices and methods.

With the development of sensors and computer technology, three-dimensional (3D) information was involved to achieve better measuring accuracy and more detailed spatial morphology of plant. Stereo vision is one of the most commonly used techniques for plant growth measurement [2,3,4,5,6]. The method usually has fine resolution and good measuring speed but its accuracy could not be so reliable since it is sensitive to the texture of measured object. Structured light is another technique to acquire 3D information and it has been successfully used in high-accurate plant growth monitoring [7] and other related studies [8,9,10], but the equipment is either complicated or expensive, which could limit its application. Time-of-flight (ToF) cameras were also introduced by some studies for 3D plant measurement [11,12,13,14]. High measuring speed and strong robustness are the advantages of these devices, whereas the major weakness is the existing high noise among 3D data. In addition, plant 3D information can be acquired from 2D images [15] or videos [16,17] captured by a single camera. There are also many cost-intensive methods focusing on detailed 3D reconstruction for plants using devices such as laser scanners [18,19] and light-filed cameras [20], or studying plant internal microstructure in 3D with tomography technology [21,22,23].

In recent years, consumer-grade depth (or known as RGB-D) sensors have drawn much attention from researchers of different fields, due to their low price, high frame rate and strong robustness. Besides, these sensors usually have an integrated color camera, which makes it possible to acquire color and 3D information simultaneously. Kinect is one of the most representative depth sensors. The first generation of Kinect (v1), released in 2010 by Microsoft, uses structured light (light coding) to acquire 3D information and it has been introduced in plant growth measurement by many researchers, such as [24,25,26]. While the newer version (v2) of Kinect (released in 2014), based on ToF measuring principle (using infrared light with a wavelength of 860 nm [27]), has a higher resolution and better accuracy than Kinect v1. This brings better opportunities for plant growth measurement study. A few valuable attempts have been carried out based on Kinect v2, including open field crop monitoring [28,29,30,31], greenhouse plant measurement [32], apple trees modeling [33], etc. However, the existing high noise of Kinect-measured point cloud [34] is still a challenge for close-range plant measuring and modeling. Although some robust modeling algorithms have been proposed, such as KinectFusion [35,36], the results of plant 3D reconstruction are not always satisfactory [37].

The paper proposes an automatic system to perform 3D reconstruction and measure key growth parameters for single potted leafy vegetables, based on Kinect v2. The goal is to obtain fine 3D point clouds and mesh models of plants, and estimate height, (total and projected) leaf area, volume and biomass (fresh/dry weight), from the measured 3D data, with good accuracy and efficiency. Lettuce was used as the main research object in our study.

The major contribution of this study are as follows. First, a non-destructive plant 3D measuring system was built based on a low-cost sensor, and the scanning and data analyzing processes work automatically using a few suitable algorithms. Second, the system can achieve fine 3D reconstruction for leafy vegetables, while measuring the key growth parameters accurately. Besides, the system shows good robustness and suitability for different leafy vegetables and it has the potential for future applications such as plant growth monitoring.

## 2. Materials and Methods

### 2.1. Platform

**Figure 1:**
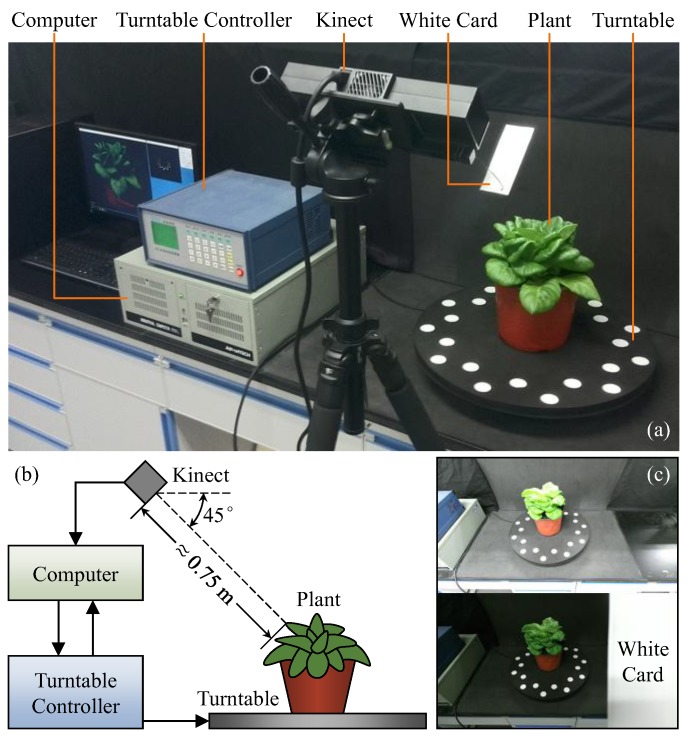
Platform of our system (**a**), relationship of each component (**b**) and effect of the white card (**c**). The arrows in (**b**) represent data flow directions.

As shown in Figure 1a, the platform of our system mainly consists of a Kinect v2 sensor, a computer, a turntable with markers and a turntable controller. The computer was used to acquire and process the data captured by Kinect and control the turntable by communicating with the turntable controller. The measured plant was placed on the center of turntable. The Kinect was mounted on a tripod, facing the plant downward by 45°, at a distance of about 0.75 m (Figure 1b). The background was built with black materials to avoid environmental disturbance.

To obtain fine color images, a white card was used to balance color from outside because the integrated color camera of Kinect is fully automatic and non-adjustable. The card was bound with Kinect on the right side near its color camera, occluding one third of the camera view. As shown in Figure 1c, the image without the white card is so bright (due to the black scene) that the plant color is seriously distorted, whereas the image with the card appears normal. Besides, the measured region was not hidden by the card for the depth camera since it is on the left of the color camera.

### 2.2. Data Acquisition

**Figure 2:**
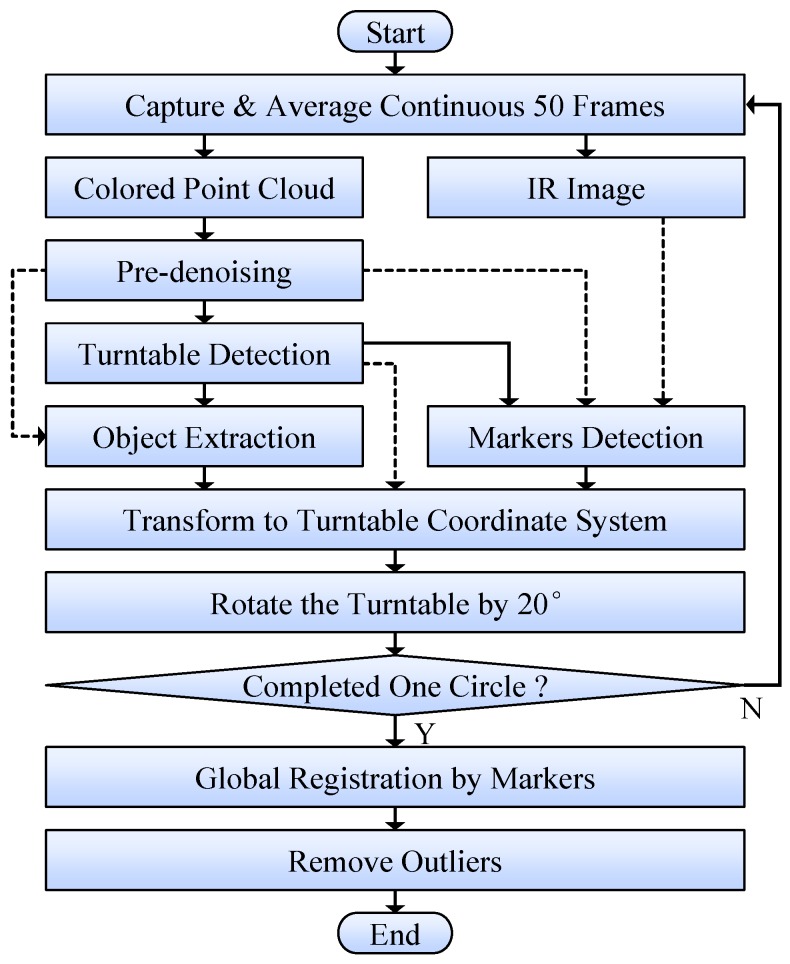
Data acquisition procedure of our system. Solid lines are the main processes and dashed lines represent to use the information/data obtained by previous steps.

The procedure of data acquisition based on our platform is shown in Figure 2, which was automatically controlled by the computer. Each measured plant was scanned from 18 views by turning the turntable every 20° The turntable stopped every time after each turn, so that the measured object can be relatively static for Kinect to acquire data.

For each time of data acquisition, 50 continuous frames were captured and averaged to decrease random noise. The obtained point cloud was colored by mapping color image onto 3D points using the interfaces provided by Kinect SDK. As result, a colored point cloud and an IR image were obtained for each view (since Kinect’s depth camera can capture point cloud and IR image simultaneously). The point cloud was pre-denoised first to remove flying pixels and violently-fluctuating points. Then the turntable was detected from the point cloud by plane fitting and circle recognition algorithms. Based on the detected turntable information (orientation, inlier indexes, etc.), the measured object was extracted from point cloud, meanwhile markers were detected from IR image and their 3D coordinates were also obtained from the point cloud. After that, the extracted object point cloud and marker points were transformed to the turntable coordinate system, based on turntable orientation and turning angle. The turntable coordinate system was defined as: the origin is the turntable center; the *z* axis points upward, perpendicular to the turntable; and the *y* axis points to the projection of the first viewing direction on *z* = 0 plane.

**Figure 3:**
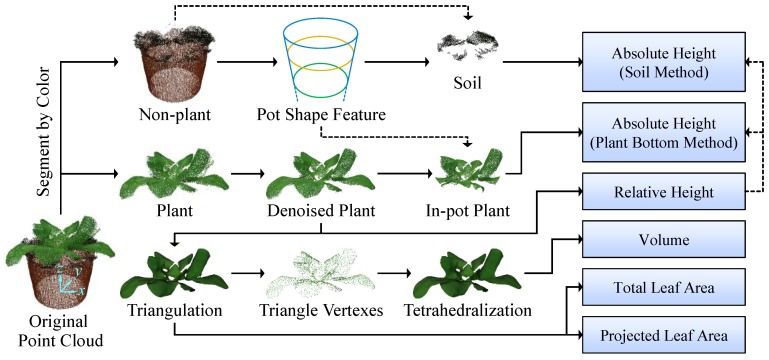
Main procedure of point cloud processing and plant growth parameters measurement. Solid lines are the main processes and dashed lines represent to use the information/data obtained by previous steps. The point clouds and triangular meshes appeared in this figure can be found in Supplementary Material A.

After all the point clouds from 18 views were obtained, global registration was performed with the clouds’ corresponding marker points, based on the iterative closest point (ICP) algorithm [38]. At last, outliers were removed from these point clouds by neighbor-checking, so that seriously deviated noisy points can be eliminated. The processed 18 point clouds can be seen as a whole in Figure 3 (bottom-left), with their common coordinate system.

Based on this principle, it is neither necessary for Kinect to be fixed at a certain position, nor a calibration was needed before each scan, since the turntable and markers were detected dynamically. Therefore, the system robustness can be guaranteed and it is easy to use.

### 2.3. Plant and Non-Plant Segmentation

The point clouds of potted plants include plant and non-plant parts. As shown in Figure 3, the plant part is the major data used to measure growth parameters and the non-plant part is used to obtain information for absolute height measurement. Therefore, an accurate segmentation is needed for both the plant and non-plant point clouds.

The segmentation is based on color information, since the plant part is basically green and it can be obviously distinguished from the non-plant part. However, the color of each local area is not so coherent due to the inconsistent color mapping of different views’ point clouds. This brings difficulty for accurate segmentation. Therefore, the local color was smoothed first using a neighbor-based weighting method. But the smoothed color was only used as reference for segmentation, rather than changing the color of the actually processed points, so that color details can be kept for other purposes (though it was not involved in the current study).

**Figure 4:**
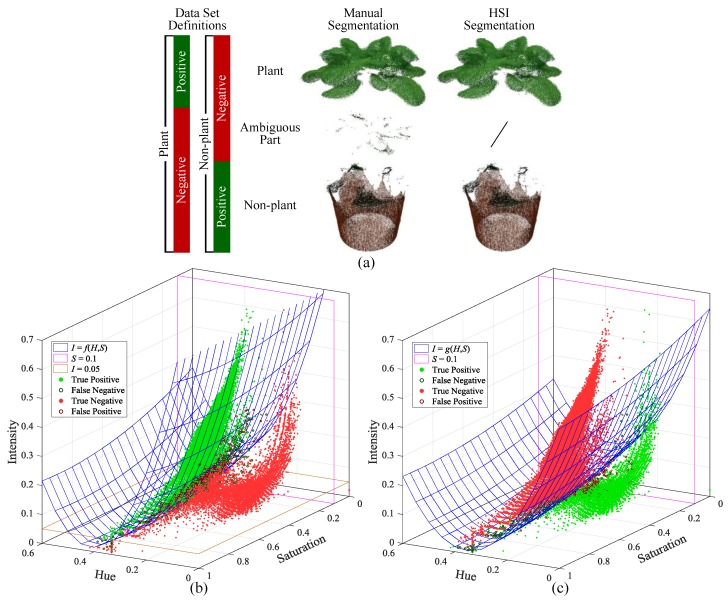
Plant and non-plant segmentation based on HSI color space: (**a**) data set definitions and segmentation results; (**b**) plant segmentation model; and (**c**) non-plant segmentation model. The data points in (**b**,**c**) are from the training set (combined by the data of three different sized plants). The interactive 3D scatter plots of (**b**,**c**) can be found in Supplementary Material B.

After that, the plant and non-plant segmentation was performed based on HSI (Hue-Saturation-Intensity) color space, which is commonly used for color-based segmentation since it is more intuitive than RGB (red-green-blue) color model and can be efficiently converted from RGB values [39]. To make the segmentation reliable and robust sufficiently, we selected three pots of lettuce in different sizes and segmented their point clouds manually to get the HSI distribution (Figure 4), so that these data can be used as training set to build segmentation models. During manual segmentation, a few points were treated as ambiguous part, because the color of these points seems like neither plant nor non-plant. The reason for this is to build two different models for plant and non-plant segmentations respectively, in order to get clean point clouds with few ambiguous points. Thus, the ambiguous part was regarded as negative sample for both the plant and non-plant parts (Figure 4a). Based on the manually segmented samples (Figure 4b,c), quadric surfaces were constructed for the segmentation, which were obtained by polynomial fitting with the manually-selected points around the boundaries between positive and negative samples. However, we cannot find a surface to separate the two kinds of samples perfectly, so extra thresholds were used along with the constructed surfaces. The resulting segmentation models are as follow. Note that *H*, *S* and *I* channels have been normalized into [0, 1), [0, 1] and [0, 1], respectively.

Plant:

(1) $$\begin{array}{c} S>0.1\;\mathrm{and}\;I>0.05\;\mathrm{and}\;I\geq f(H,S),\\ f(H,S)=1.114-4.9H-0.229S+6.066H^{2}-0.1638H\cdot S+0.1852S^{2}, \end{array}$$

Non-plant:

(2) $$\begin{array}{c} S\leq 0.1\;\mathrm{or}\;I\leq g(H,S),\\ g(H,S)=0.6475-2.777H-0.2797S+3.714H^{2}+0.1326H\cdot S+0.1133S^{2}, \end{array}$$

where *f*(*H*, *S*) and *g*(*H*, *S*) are the functions of the quadric surfaces for plant and non-plant segmentations respectively.

The result of HSI-based segmentation can be seen in Figure 4a, which shows little difference comparing to the manually segmented result. To future evaluate the effectiveness of this method, another three pots of lettuce in different sizes were used as test sets. The reference data of test sets were also segmented manually. Table 1 lists the HSI-based segmentation results of the training set and test sets, using true positive rate (TPR) and true negative rate (TNR) as index (calculated by points number). It shows that all the TPRs and TNRs are above 99% for both the plant and non-plant segmentations and there is no obvious difference between training set and test sets. Actually, the error was mainly from the ambiguous points around the quadric surfaces (see the circled points in Figure 4b,c), which has little effect on the segmented result. Therefore, it indicates that the method is effective enough for our research objects.

### 2.4. Plant Point Cloud Processing

#### 2.4.1. Noise Reduction

Since the plant point cloud has been obtained, future processing including the measurement of most plant growth parameters, can be performed on this basis. As shown in Figure 3, the first step is to reduce noise for the plant point cloud, because future processes could be affected if the noisy points of leaves were not eliminated. The noise is not only located around the leaf surfaces but also caused layered points for some leaves. Our solution used different algorithms to reduce these two kinds of noise respectively.

**Figure 5:**
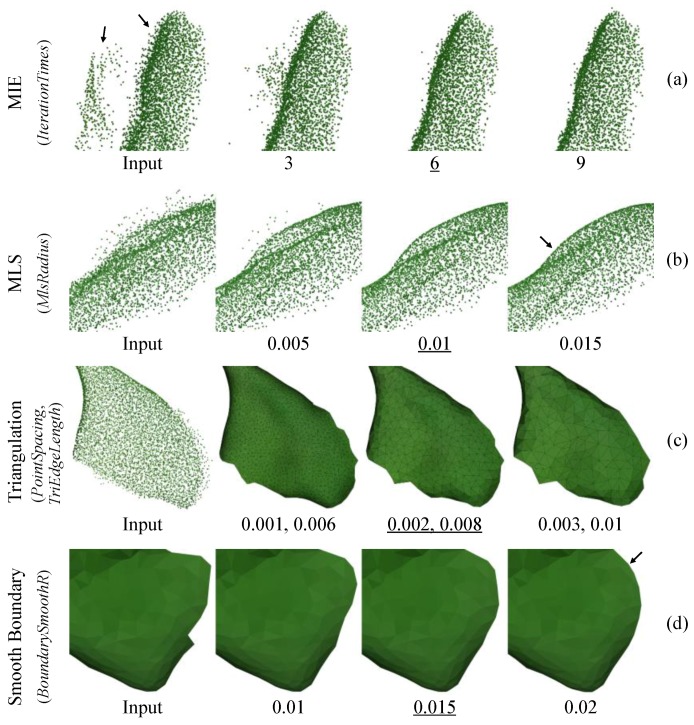
Key parameter optimization for major algorithms. In each row (**a**–**d**), the left title is the algorithm name with its key parameter(s) in parentheses, then follows the input data and the results under three different settings, where the optimized values are underlined. Arrows indicate the discussed key areas. All numbers are in meters except for *IterationTimes*.

First, the multi-view interference elimination (MIE) algorithm was used to process the layered points. In Figure 5a, the two layers of points were actually from one leaf, because the refraction, transmission or multi-reflection of the ToF measuring light inside/among leaves resulted in the inaccurate distance measurement, thereby leading to separated layers when a leaf was measured from different sides. The *IterationTimes* is a key parameter of MIE to limit its max iteration times, since MIE is a time-consuming iterative method. The optimal value for the parameter is 6 since it can well recover two layers into one layer, whereas the result of 3 (times) is unsatisfactory and a large value (9 times) is unnecessary due to time-saving purpose.

After that, the moving least squares (MLS) algorithm [40] was used to reduce the remaining local noise around leaf surfaces. *MlsRadius* is a key parameter of MLS, which represents the radius of smoothing range. Here 0.01 m was selected for *MlsRadius* to achieve satisfactory result (Figure 5b), because some noise still exist when using a smaller value (0.005 m), whereas points could be over-smoothed if a larger value (0.015 m) was given. So far, the denoised plant point cloud is clean and smooth enough to be applied in later processes.

#### 2.4.2. Triangulation

To represent leaf surfaces, triangular mesh was constructed based on the denoised plant point cloud. Since the cloud is too dense to generate triangles, a down-sampling was performed first by repeatedly merging neighborhood points until any two points’ distance is greater than or equal to the given threshold *PointSpacing*. This ensures proper space among the down-sampled points to prevent generating abnormal triangles.

After that, triangulation was performed by the following steps: First, generate triangles from the down-sampled points by ensuring that no point is in the minimum enclosing ball of each triangle (similar to the Delaunay triangulation [41]) and all triangle edges should be smaller than the threshold *TriEdgeLength* to avoid connecting discontinuous surfaces. Second, apply filters to remove abnormal triangles, including redundant paired triangles, suspended triangles, multi-connected triangles and the triangles nearly perpendicular to surfaces. Third, remove small components (i.e., the interconnected triangles with a small total area). Fourth, generate triangles to fix the holes with just three edges.

A triangular mesh with few defects can be obtained after these steps. Figure 5c shows the effects of different *PointSpacing* and *TriEdgeLength*, which are used to control the fineness of triangular mesh and should be adjusted together since they are associated by scale. Here we select 0.002 m and 0.008 m for the two parameters respectively, because the leaf details were weakened when using larger values (0.003 m, 0.01 m), whereas smaller values (0.001 m, 0.006 m) are unnecessary thereby avoiding generating too many triangles and slowing down later processes.

However, the constructed triangular mesh has uneven boundaries that may result in inaccurate leaf measurement. The jagged edges (Figure 5d) were mainly caused by the noise on leaf boundaries, which cannot be properly processed by the abovementioned point-cloud-based denoising algorithms. Therefore, boundary smoothing was performed by moving each boundary point and its neighbors to their weighted-average positions based on local computations. *BoundarySmoothR* is the threshold to represent smoothing range. Here 0.015 m was selected for the parameter, because sharp edges could still exist when using a smaller value (0.01 m), whereas a larger value (0.02 m) may result in over-smoothed boundaries and leaf surface distortion. Ultimately, a smoothed triangular mesh can be obtained.

### 2.5. Segmentation Based on Pot Shape Feature

**Figure 6:**
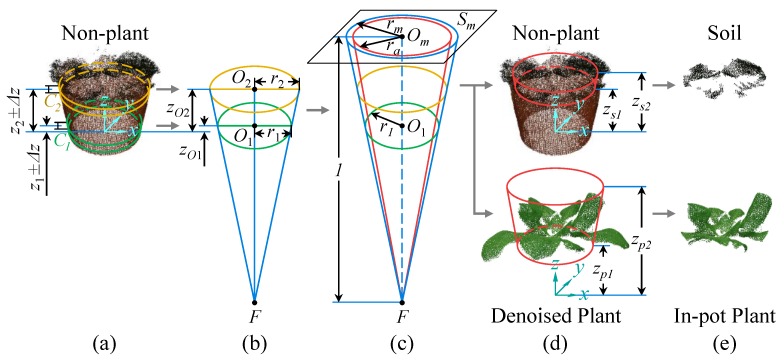
Principle of pot shape feature extraction and in-pot parts segmentation: (**a**) sample points extraction; (**b**) circle fitting and cone apex calculation; (**c**) construction of datum plane and adjusted cone (red); (**d**) in-pot point clouds segmentation; (**e**) segmented results.

The in-pot parts (including soil and in-pot plant), which are necessary for plant height measurement (Figure 3), need to be obtained by pot-shape-based segmentation. So, the relevant pot shape information should be acquired first. As shown in Figure 6, the pot shape can be represented as a part of a cone, since the pot we used is basically truncated-cone-shaped. The first step is to extract two sets of points (*C*_1_ and *C*_2_) as samples from the non-plant point cloud at two different heights, within the intervals of *z* coordinate (Figure 6a): *z*_1_ ± Δ*z* for *C*_1_ and *z*_2_ ± Δ*z* for *C*_2_.

Then, two circles were fitted by least square method (in 2D space) based only on the (*x*, *y*) coordinates of the points in *C*_1_ and *C*_2_, respectively (Figure 6b). The fitted circle radiuses are *r*_1_ and *r*_2_. The circle centers (in 2D) were used as the (*x*, *y*) coordinates of their 3D centers *O*_1_ and *O*_2_, while the *z* coordinates of *O*_1_ and *O*_2_ are the average *z* of *C*_1_ and *C*_2_, respectively.

Therefore, the two 3D circles can be obtained, which represent the horizontal sections of the pot at two different heights. Based on this, a cone was then constructed (Figure 6b) and its apex *F* can be calculated as follow:

(3) $$F=(O_{1}-O_{2})\cdot \frac{r_{2}}{r_{2}-r_{1}}+O_{2}$$

Since the cone is roughly tangent to the pot surface, it can be used to segment the in-pot points. To ensure the robustness of segmentation, an adjusted cone was constructed by shrinking the original cone (Figure 6c), so that no points near the pot side surface would be involved. Moreover, to simplify computation for further segmentation, a datum plane *S_m_* was constructed at a vertical distance of 1 m above the apex *F*. On this plane, the radius *r_a_* of the adjusted cone’s section can be calculated using *O*_1_ and *r*_1_:

(4) $$r_{a}=\eta \cdot r_{m}=\eta \left|\frac{r_{1}}{z_{O1}-z_{F}}\right|,$$

where *η* is the shrinking coefficient of the adjusted cone, *r_m_* is the radius of the original cone section on *S_m_*, *z_O_*_1_ and *z_F_* are the *z* coordinates of *O*_1_ and *F* respectively.

When a point is projected onto *S_m_* by conical projection, its *x* and *y* coordinates can be calculated as follow:

(5) $$\{\begin{array}{l} px(Q)=x_{F}+\frac{x_{Q}-x_{F}}{z_{Q}-z_{F}}\\ py(Q)=y_{F}+\frac{y_{Q}-y_{F}}{z_{Q}-z_{F}} \end{array},$$

where *Q* is the point to be projected, *px*(*Q*) and *py*(*Q*) are the functions to get the *x* and *y* coordinates of *Q*’s projection respectively, (*x_Q_*, *y_Q_*, *z_Q_*) and (*x_F_*, *y_F_*, *z_F_*) are the (*x*, *y*, *z*) coordinates of *Q* and *F* respectively.

Thus, the center *O_m_* of the adjusted cone’s section can be expressed as (*px*(*O*_1_), *py*(*O*_1_), *z_F_* + 1), by projecting *Q*_1_ onto *S_m_* (Figure 6c). Based on this principle, the segmentation can be performed by checking whether a point’s projection on *S_m_* is in the circle with center *O_m_* and radius *r_a_*, combining with additional *z* coordinate thresholds (Figure 6d). The equations for soil and in-pot plant segmentation can be expressed as follow:

(6) $$C_{S}=\left\{\begin{array}{ccc} Q & | & \sqrt{{\left[px(Q)-x_{Om}\right]}^{2}+{\left[py(Q)-y_{Om}\right]}^{2}}<r_{a},\;z_{Q}\in [z_{s1},z_{s2}],\;Q\in C_{NP} \end{array}\right\},$$

(7) $$C_{IPP}=\left\{\begin{array}{ccc} Q & | & \sqrt{{\left[px(Q)-x_{Om}\right]}^{2}+{\left[py(Q)-y_{Om}\right]}^{2}}<r_{a},\;z_{Q}\in [z_{p1},z_{p2}],\;Q\in C_{DP} \end{array}\right\},$$

where *C_S_* and *C_IPP_* are the point clouds of soil and in-pot plant respectively, *Q* is a point in the non-plant point cloud (*C_NP_*) or the denoised plant point cloud (*C_DP_*), *x_Om_* and *y_Om_* are the *x* and *y* coordinates of *O_m_* respectively, [*z_s_*_1_, *z_s_*_2_] and [*z_p_*_1_, *z_p_*_2_] are the intervals of *z* coordinate for soil and in-pot plant segmentation, respectively.

For our experimental objects, the values of the abovementioned parameters are: *z*_1_ = 0.035 m, *z*_2_ = 0.095 m, Δ*z* = 0.005 m, *η* = 0.9, *z_s_*_1_ = 0.11 m, *z_s_*_2_ = 0.168 m, *z_p_*_1_ = 0.12 m, *z_p_*_2_ = 1 m. The segmented results can be seen in Figure 6e.

### 2.6. Growth Parameters Measurement

#### 2.6.1. Height

**Figure 7:**
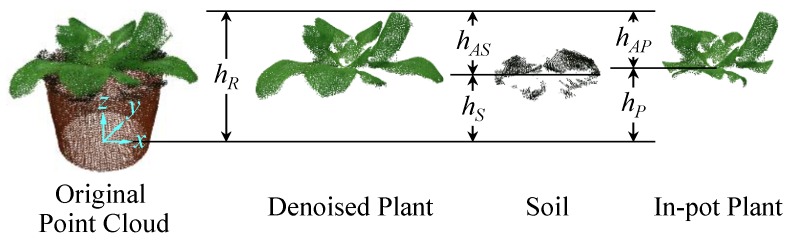
Definitions and principles of plant height measurement.

Since all the required data have been obtained by previous processes, plant growth parameters can be measured on this basis. Plant height was measured based on the point clouds of denoised plant, soil and in-pot plant. Relative height and absolute height(s) were measured in this study, whose definitions can be seen in Figure 7.

Relative height (*h_R_*) was defined as the vertical distance from pot bottom to the top of plant. Since the coordinate origin of the processed point clouds is exactly on the turntable surface, the *z* coordinate of pot bottom can be regarded as 0. Therefore, *h_R_* can be obtained simply by getting the maximum *z* coordinate of the denoised plant point cloud.

Absolute height represents the vertical distance from the base of main stem to the top of plant, which can be measured by two different methods: soil method and plant bottom method. The soil method regards the soil surface as the (vertical) location of stem base. So, the absolute height of the soil method (*h_AS_*) is the difference between the relative height (*h_R_*) and soil height (*h_S_*):

(8) $$h_{AS}=h_{R}-h_{S}$$

where *h_S_* is the average *z* coordinate of the soil point cloud.

However, soil could be totally hidden by leaves, so there may be less or no points in the soil point cloud. Therefore, to ensure the reliability of soil height measurement, a threshold *MinSoilPoints* was used to limit the minimum allowable number of soil points. That is to say, if the number of soil points is smaller than the threshold, the calculation of *h_AS_* will not be performed. *MinSoilPoints* was set to 10 in our experiments.

The plant bottom method regards the lowest point of the in-pot plant as the stem base of plant. The reason of using in-pot plant rather than the whole plant is to avoid involving the outside leaves, which may have lower points than the inside part. Thus, the absolute height of the plant bottom method (*h_AP_*) can be calculated as follows:

(9) $$h_{AP}=h_{R}-h_{P},$$

where *h_P_* is the minimum *z* coordinate of the in-pot plant point cloud.

#### 2.6.2. Leaf Area

**Figure 8:**
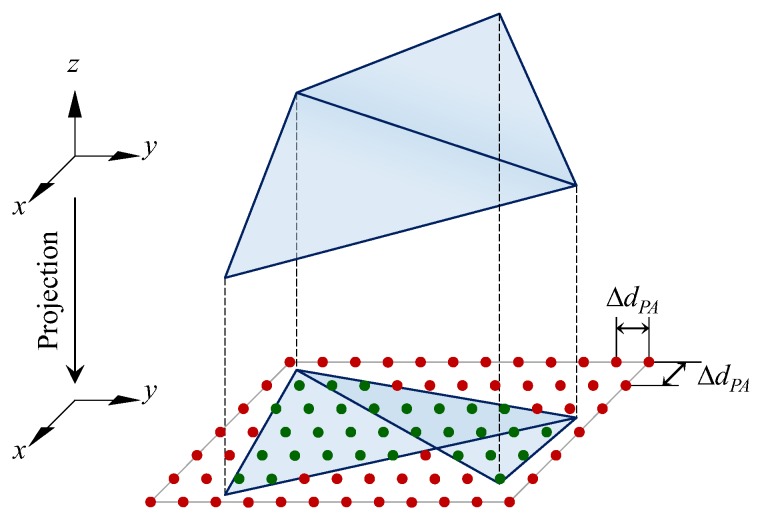
Principle of projected leaf area measurement. The points inside and outside the projected triangles are in green and red, respectively.

Since leaf surfaces have been represented by the triangular mesh, total leaf area and projected leaf area can be measured based on the mesh. In our study, total leaf area was simply calculated by summating the area of all triangles in the mesh. While projected leaf area was defined as the vertical projection area of the measured plant, thus the triangular mesh was projected onto the *x*-*y* plane along *z* axis to get the area. However, the projected triangles could be overlapped (such as the case in Figure 8) and the combination for large number of triangles can be a complex process. Therefore, a simple but not completely precise method was used to get the projected area. As shown in Figure 8, a matrix of points with a same spacing Δ*d_PA_* in *x* and *y* direction was created on the projection plane first. Then check whether each point was inside any of the projected triangles. So, the projected leaf area (*S_PA_*) can be calculated based on the number of the inside points:

(10) $$S_{PA}=N_{PA}\cdot \Delta d_{PA}^{2},$$

where *N_PA_* is the number of the points inside any of the projected triangles.

**Figure 9:**
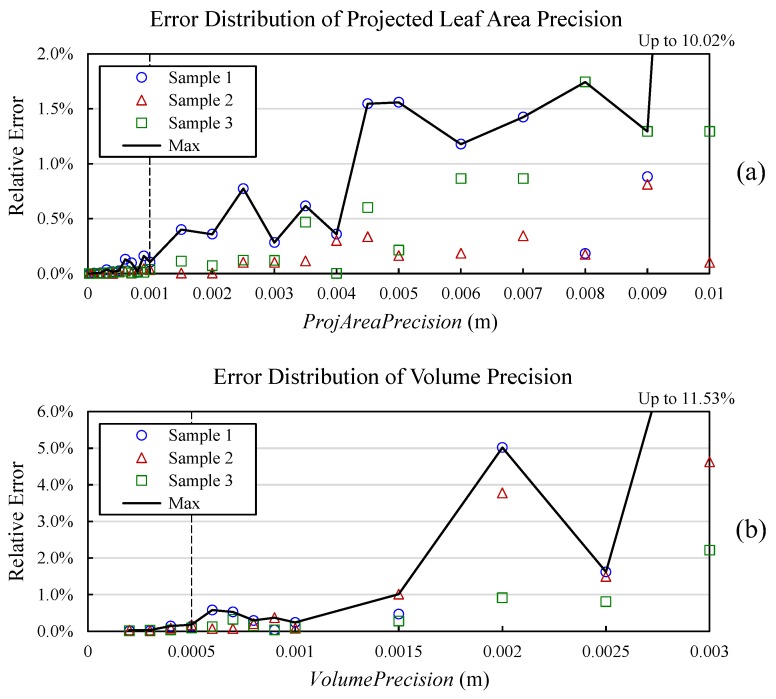
Precision parameters optimization for projected leaf area (**a**) and volume (**b**). The selected values are shown as dashed lines.

Actually $\Delta d_{PA}^{2}$ is the area represented by each matrix point and Δ*d_PA_* can be seen as the precision of area calculation. So, the resulting *S_PA_* can be roughly equal to the precise projected area if Δ*d_PA_* is small enough. *ProjAreaPrecision* is the parameter to determine the length of Δ*d_PA_*. As shown in Figure 9a, three different sized plants (lettuce) were used as samples to explore the effect of different precisions. The results under a small-enough *ProjAreaPrecision* (0.0001 m) were used as reference for error calculation. The figure shows that the error almost stopped decreasing when the parameter was set smaller than 0.001 m, meanwhile the max error (<0.2%) is acceptable. So, 0.001 m was selected for *ProjAreaPrecision* and the resulting projected leaf area can be regarded precise enough.

#### 2.6.3. Volume

**Figure 10:**
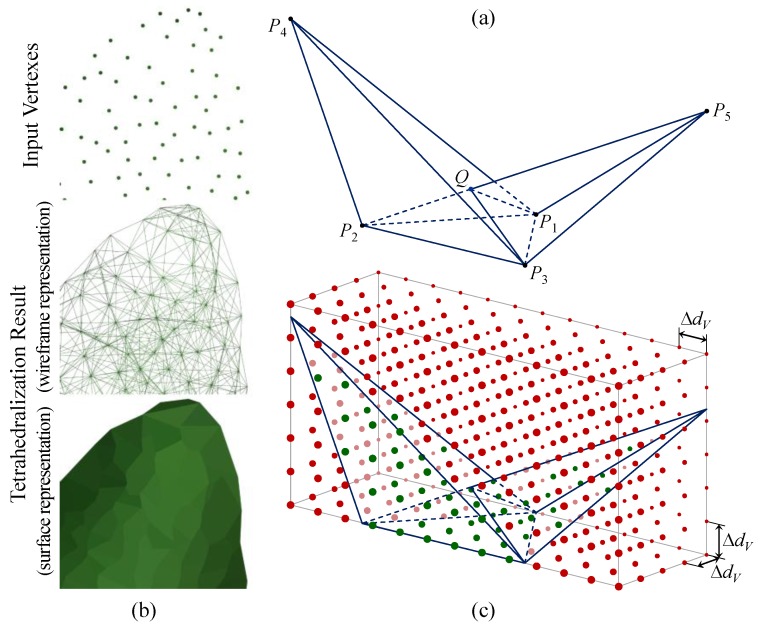
Principle of tetrahedralization and volume measurement: (**a**) overlapped tetrahedrons construction; (**b**) tetrahedralization result; (**c**) volume measuring principle. In (**c**), the points inside and outside the tetrahedrons are in green and red, respectively, and their sizes are differed by viewing distance (the farther the smaller).

The volume of plant was measured based on tetrahedrons. Similar to the triangulation process, the tetrahedralization should be based on a down-sampled point cloud to construct tetrahedrons with proper sizes. Since the triangulation had achieved a smooth and fine result, the triangle vertexes extracted from triangular mesh were used as the points to construct tetrahedrons (Figure 3). Then, overlapped tetrahedrons were constructed from these points and the maximum edge length of tetrahedrons was limited by the threshold *TetraEdgeLength*. That is to say, all tetrahedron edges should be smaller than the threshold. The reason of generating overlapped tetrahedrons, rather than using a subdivision method, is to involve all points and represent the actual leaf shape as fully as possible. As shown in Figure 10a, *P*_1_ to *P*_5_ are the points to construct tetrahedrons and the distance between any two points is smaller than *TetraEdgeLength*, except for *P*_4_ and *P*_5_. Thus, two overlapped tetrahedrons (*P*_1_*P*_2_*P*_3_*P*_4_ and *P*_1_*P*_2_*P*_3_*P*_5_) can be constructed (*Q* is their intersection). So, if the overlapping case is not allowed, only one tetrahedron can be constructed from four of the five points (*P*_1_*P*_2_*P*_3_*P*_4_ as example) and as result, another point (*P*_5_) will be useless. Therefore, our method can estimate the volume of measured object from points more reasonably. Besides, to ensure the consistency of shape representation, *TetraEdgeLength* was set to the same value (0.008 m) as *TriEdgeLength*.

The close-up tetrahedralization result of a leaf (Figure 10b) shows that even a small number of points can construct large numbers of tetrahedrons. Therefore, it is not easy to get the (non-repetitive) total volume from these overlapped tetrahedrons. So, we used a method similar to the projected leaf area measurement, to measure the volume with 3D point matrix. As shown in Figure 10c, the matrix points have a same spacing (Δ*d_V_*) in *x*, *y* and *z* direction. Thus, the volume (*V*) can be calculated based on the number of points (*N_V_*) inside (any of) the tetrahedrons:

(11) $$V=N_{V}\cdot \Delta d_{V}^{3}$$

Similarly, $\Delta d_{V}^{3}$ is the volume represented by each matrix point. *VolumePrecision* is the precision parameter to determine the length of Δ*d_V_*. As shown in Figure 9b, the same three samples used for *ProjAreaPrecision* optimization were also used to optimize *VolumePrecision* and the results under 0.0001 m were used as reference for error calculation. It shows that the error almost stopped decreasing when the parameter was smaller than 0.0005 m, while the max error (<0.2%) is also acceptable. So, 0.0005 m was selected for *VolumePrecision* to ensure that the volume calculation is precise enough.

### 2.7. Experimental Design

To verify the accuracy and effectiveness of the proposed system and methods, experiments were carried out in batches. Potted lettuce (variety: *Qingfeng*, a kind of butter lettuce) was used as major experimental object. The lettuce seeds were planted on the same day and each pot was ensured to have only one lettuce before experiment. 7 batches of experiments were carried out once a week, started form the 4th week (day 28) after sowing and each batch used 9 pots of lettuce, thus there are 63 samples in total and the time span of measurement is 43 days (from day 28 to day 71). The reason for this is to acquire and analyze the data of different sized plants, but no experiment was arranged in the first 4 weeks because the plants were too small for Kinect to obtain valid data. In the experiment, each plant was measured first by our system (under the same indoor lighting condition), then its height and projected leaf area were measured by other non-destructive methods as reference. After that, the leafy part was cut off and destructive methods were then applied to measure other reference growth indexes, sequentially, including fresh weight, total leaf area, volume and dry weight. That is to say, plants in different growth stages were measured by our system and reference methods, and each plant was measured only once as the data of one sample.

The program used to obtain and process point clouds was written in C++, based on the Point Cloud Library (PCL) v1.7.2 [42]. And all the above mentioned algorithms have been integrated into the program, so that it can run automatically once all parameters have been set properly. The key parameters and their values used in our experiment are listed in Table 2. Besides, all data were processed on a computer (the one in Figure 1) with Intel Core i7-4790 CPU and 8 G DDR3 RAM (GPU was not involved in computation).

## 3. Results and Discussion

### 3.1. Height Measurement

As mentioned above, the relative and absolute heights were measured by our system and reference method. The reference heights were measured by hand with a ruler, where the relative height was measured vertically from pot bottom to the top of plant and the absolute height was measured from soil to the top of plant. The manually measured absolute height was used as reference for both soil and plant bottom methods.

**Figure 11:**
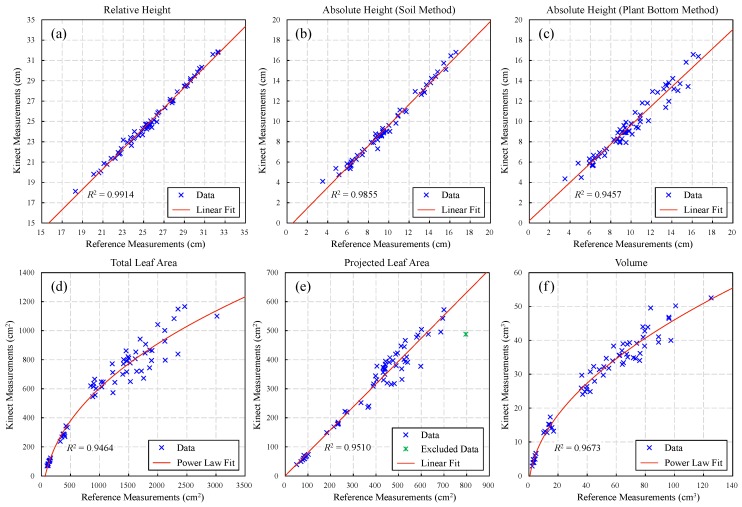
Data distribution and fitting results for relative height (**a**), absolute height based on soil method (**b**) and plant bottom method (**c**), total leaf area (**d**), projected leaf area (**e**) and volume (**f**). The original data for this figure can be found in Supplementary Material D.

The result of height measurement for the 63 samples is shown in Figure 11a–c. It shows that the Kinect measurements have good linear relationship with reference data, for all the three measured results. Therefore, linear fitting was performed for these results and the corresponding fitting details can be seen in Table 3. It shows that the fitting goodness of relative height (*R*^2^ = 0.9914) is better than the two absolute heights, while the soil method (*R*^2^ = 0.9855) performed better than the plant bottom method (*R*^2^ = 0.9457) for absolute height. However, since the soil of some samples was barely visible for Kinect, there are only 57 valid data points in the result of soil method.

Mean absolute error (MAE) and mean absolute percentage error (MAPE) were used in Table 4 to show the accuracy of the Kinect measurements comparing to reference values for the data with linear relationships. All the three kinds of measured heights show good accuracy, where the relative height has the lowest MAPE (2.58%) and the soil method (absolute height) has the lowest MAE (0.4657 cm). For the relative height result, the existing error was mainly caused by random factors since the slope (1.002) of the fitted line is close to 1, but its intercept (−0.7111) could result from system error. For the result of absolute height with soil method, the main error source is the uneven soil surface, which could bring errors for both Kinect and manual measurements. For the result of plant bottom method, the error was mainly caused by the occlusion of leaves, because the actual bottom of the in-pot plant could be hidden by leaves.

As a comparison, the absolute height has advantage in reflecting actual plant height, despite the better linear relationship of the relative height measurement. For the absolute height measurement, the soil method is more accurate than the plant bottom method, but the latter can be used as an alternative when the former cannot obtain valid data due to unobservable soil.

### 3.2. Leaf Area Measurement

To verify the accuracy of the (total and projected) leaf area measured by our system, reference measurement data were obtained by relatively accurate methods. The reference projected leaf area was measured by taking photos for each plant along with a few reference markers of known size, based on a color camera directly above the plant. The markers were put beside the plant, at a roughly-average height of the leafy part. So, the projected area can be calculated based on the marker area and the ratio of leaf-marker pixels (selected by hand) in the photo. The total leaf area of each plant was measured by cutting off every single leaf, and using a flatbed scanner to scan the leaves along with a few reference markers of known size. So, the area of scanned leaves can be calculated similarly, based on marker area and the leaf-marker pixel ratio in the scanned images. Thus, for each plant, the sum of all leaves’ scanned area can be seen as the total leaf area.

**Figure 12:**
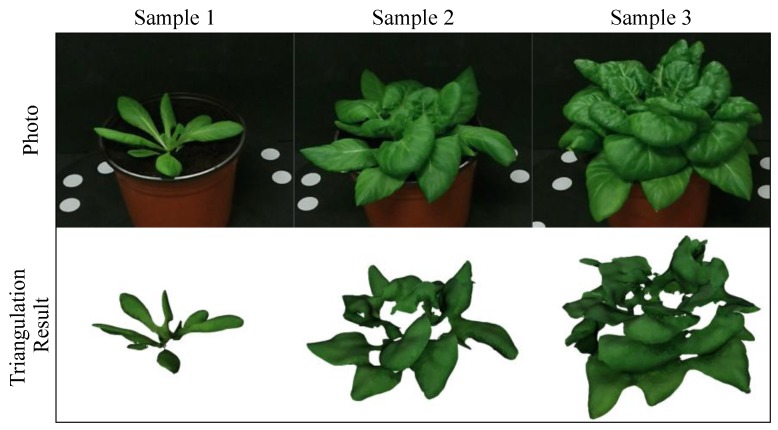
Triangulation results of plants in different sizes. The triangular meshes can be found in Supplementary Material C.

The result of total leaf area measurement (Figure 11d) shows that the reference-Kinect measurements generally follow a power law distribution. Besides, the data points became discrete as the total area increased. The reason is that the occlusion of leaves appeared only when the plant had grown to a certain stage and the individual differences of leaf occlusion resulted in the discrete data. This can also explain why the data points follow a power law distribution, because when a plant had more leaves, the more parts of leaves were invisible for Kinect. Figure 12 shows the triangulation results of three different sized plants. It is obvious that, as new leaves grew, the inside leaves were hidden by the outside ones, so the resulting triangular mesh can only represent the visible leaves. Another reason for the missing data is that, part of leaves were either deviated from (i.e., almost parallel to) the viewing direction or hidden by other leaves during the scanning process, though these areas were visible in the photo. Besides, the completeness and correctness of the triangular mesh could be affected by many factors, including remaining noise, over denoised (removed or smoothed) points, wrongly generated triangles and so on. Nevertheless, the true total area can still be estimated from Kinect measurements, since the fitting result (Table 3) is generally good (*R*^2^ = 0.9460).

**Figure 13:**
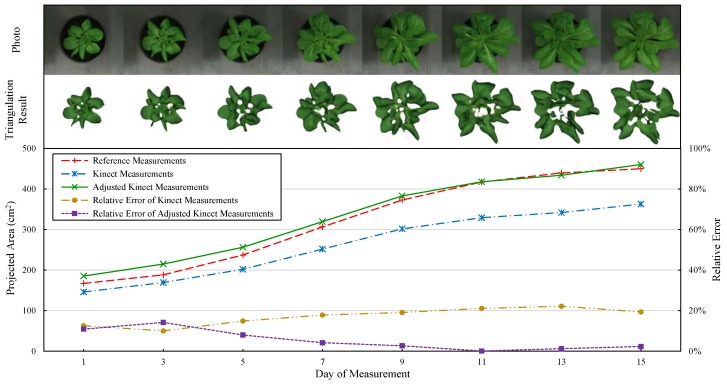
Results of projected leaf area monitoring (periodical measurement) for a single plant.

In the result of projected leaf area measurement (Figure 11e), the data points generally have a linear relationship. There was one point excluded as abnormal data, since it has a large deviation. Similar to the total area result, the data points became discrete as the projected area increased. In addition to the effects of individual differences, the main reason is that some leaves cannot be effectively measured by Kinect even though they were visible, thereby resulting in several “holes” among leaves. This usually happened around the center of plant, especially when the plant had grown to a certain stage. Because the complicated structures of central leaves sometimes resulted in unreliable measurement due to the multi-reflection effect of ToF sensor and could be removed as noise. As can be seen from Figure 13, the triangulation results of the center leaves were not so reliable and stable, even for the different growth stages of a same plant. Besides, another error source cloud be the inaccurate measurement of leaf edges, which made some leaves a little smaller than their actual sizes. Actually, this is the reason why the fitted line (Table 3) has a small slope (0.788) and the measurement has a large error (MAPE = 21.59%) in Table 4. Overall, however, the fitting result (*R*^2^ = 0.9510) is fine enough for our system to obtain accurate projected leaf area, by adjusting Kinect measurements based on the fitted formula.

### 3.3. Volume Measurement

Similarly, the volume measured by our system was also compared with reference measurements. The reference volume was measured by water displacement method, using a measuring cylinder. Actually, to ensure accuracy, the height of liquid level was measured by taking photos and counting pixels based on the measuring cylinder’s scale. The result in Figure 11f shows that the data points follow a power law distribution. Similar to the total leaf area, this was mainly caused by the occlusion of leaves. But since the constructed overlapped tetrahedrons cannot truly reflect the thickness of leaves, the measured volume is not necessarily proportional to the true volume, as the plants grew. This cloud be another reason for the power law distribution. The error of the discrete points was possibly caused by the differences of leaf shapes, because the thickness formed by the tetrahedrons could be different on flat and uneven leaf surfaces. Overall, since the fitting result (Table 3) is fine enough (*R*^2^ = 0.9673), it is feasible to estimate true volume from Kinect measurements with good accuracy.

### 3.4. Correlations between Kinect Measurements and Biomass

To further research the correlations of Kinect measurements and biomass, fresh weight and dry weight of plant were measured during experiment. Fresh weight was measured right after the leafy part was cut off, with an electronic balance. And dry weight was measured by drying leaves with direct drying method until the samples had reached constant weight. Since biomass is usually proportional to total leaf area and volume, these two indexes measured by Kinect were used to research the correlations to fresh weight and dry weight.

**Figure 14:**
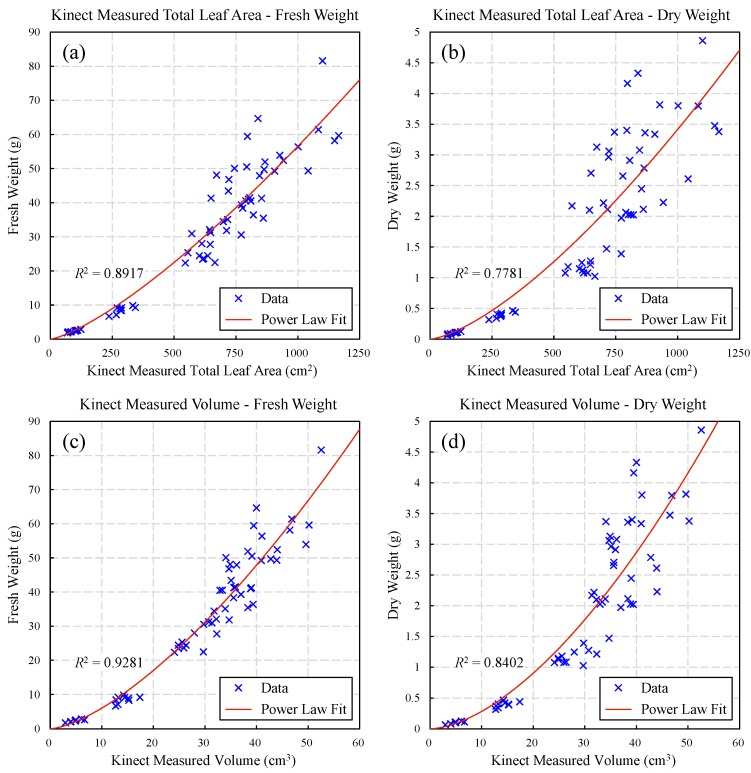
Correlations of Kinect measurements and biomass: Kinect measured total leaf area and fresh weight (**a**), Kinect measured total leaf area and dry weight (**b**), Kinect measured volume and fresh weight (**c**), and Kinect measured volume and dry weight (**d**). The original data for this figure can be found in Supplementary Material D.

The result of Kinect-measured total leaf area and fresh/dry weight can be seen in Figure 14a,b. It shows that both the two data sets follow power law distributions. Fresh weight shows better fitting goodness with total leaf area than dry weight (Table 3), but both of the two fitting results are not so satisfactory. The poor data consistency could be affected by many factors, including leaf occlusion, individual differences, biomass measuring error, old/withered leaves, etc. The individual differences of moisture content could be the reason why dry weight data are more discrete than fresh weight data, which might be caused by the different sizes or numbers of old leaves.

The data of Kinect-measured volume and fresh/dry weight also follow power law distributions (Figure 14c,d), whereas the fitting results are better than the ones of total leaf area (Table 3), for fresh and dry weight respectively. Similarly, fresh weight shows better fitting goodness with volume than dry weight and the fitting result of fresh weight is basically satisfactory (*R*^2^ = 0.9281). The error sources are generally as same as the abovementioned aspects of total leaf area. But since volume has better correlations with biomass, it indicates that the volume measurement can better reflect the actual plant biomass, than the measurement of total leaf area.

Therefore, comparing to total leaf area, the volume measured by Kinect has better correlations with biomass, while fresh weight shows better correlation with Kinect measurement than dry weight. The fitting results indicate that, it is feasible to estimate true fresh weight from Kinect volume measurement but the estimation of dry weight might not be accurate enough.

### 3.5. Performance Analysis

**Figure 15:**
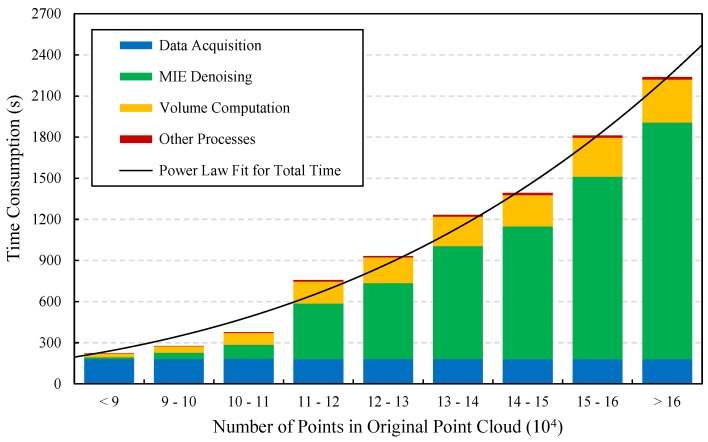
Time consumption of our system, for single plant measurement. The data are grouped by the number of points in original point clouds and the major time-consuming processes are in different colors.

To evaluate the performance of the proposed measuring system, the consumed time of each plant’s measurement was recorded. All data were obtained and processed by a same computer (mentioned in Section 2.7), and the time of data processing (not including the scanning process) was the average of three repeated tests. The statistical results of time consumption can be seen in Figure 15. The data was grouped by original point cloud size and it ranges from 3.73 min to 37.32 min. It is obvious that the total time increases with the increasing number of input points and it basically follows a power law distribution. Therefore, plant size can be a major factor that affects time consumption, thus the system efficiency will be reduced when dealing with large sized plants.

In fact, the data acquisition time basically remained unchanged (about 180 s) no matter how many points were in the point cloud, because most time was spent on the rotation of turntable. So, the increased time was mainly caused by only a few processes in the data processing stage. The figure shows that, the MIE denoising and volume computation are the major time-consuming processes, whereas the other processes contributed little to the increased time. Therefore, to reduce time consumption, the following improvements can be carried out in the future: Increase the turntable speed while ensuring the stability of plant; Optimize the MIE algorithm or replace it with other denoising methods; Decrease the precision of volume measurement to find a better balance between effect and time; And, use multithread or GPU-based methods to speed up the algorithms. As an example of multithread acceleration, the scanning process runs in one thread, while running multiple data analyzing processes for different datasets in other threads, thereby saving a tremendous amount of time (depending on CPU performance) and improving the overall throughput.

### 3.6. Future Applications

To explore the feasibility of the system for future plant growth monitoring (periodical measurement) applications, a tentative experiment was carried out by measuring a single pot of lettuce every two days (Figure 13). Since the measured plant should remain undamaged to get the data of different growth stages, we used projected leaf area to evaluate the measuring effects because the reference measurements can be obtained non-destructively. As mentioned above, the fitted formula of projected leaf area can be used to correct Kinect measurements. Thus, the result was adjusted by substituting Kinect measurements in the equation *x* = (*y* − 1.252)/0.788, which was derived from Table 3. As shown in Figure 13, the error of adjusted Kinect measurements decreased as the plant grew larger and it was almost close to zero after day 7. Whereas the (unadjusted) Kinect measurements always have large errors. This indicates that the adjustment is effective but it might not quite suitable for the plants in an early growth stage. Therefore, the overall accuracy and practicality of our system is satisfactory, which shows good feasibility for future applications such as plant growth monitoring.

**Figure 16:**
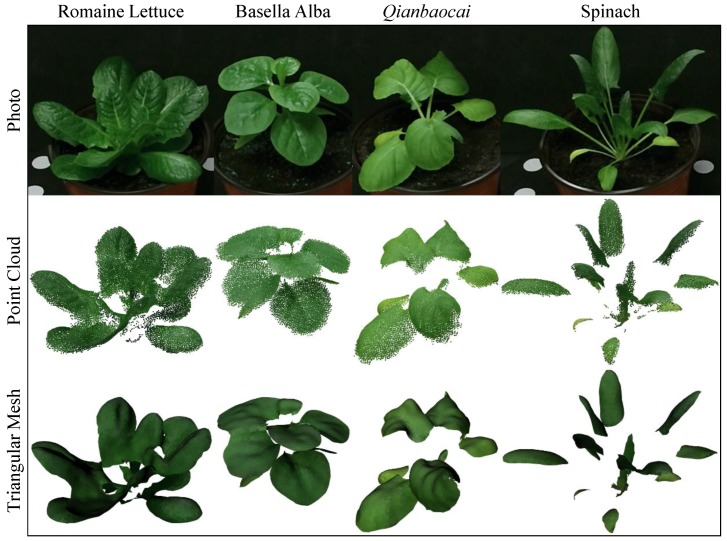
3D reconstruction results for different species of leafy vegetables. The *Qianbaocai* is a hybrid of *Brassica oleracea* L. and *Brassica campeseris* L.

In order to test the suitability of the proposed system, four different species of leafy vegetables were measured by our method, with the same settings used in the lettuce experiment. The obtained point clouds and triangular meshes can be seen in Figure 16. It shows that all the results are generally good, and both the point clouds and meshes can correctly reflect the shape of leaves. This indicates that our system has good robustness for different species of leafy vegetables and it also proves that the selected parameters are not so sensitive to different shapes of leaves. However, thin stems cannot be effectively measured by Kinect due to its limited resolution, so the stems are missing in the obtained 3D data (see the right two samples in Figure 16). This can be a limitation of our system if stem information is needed. Besides, the current HSI segmentation model is not necessary suitable for non-green vegetables (e.g., in purple), thus an adjusted model is required in such situation. Nevertheless, the overall robustness of the proposed method can be guaranteed and it shows good practicality for the measurement of different leafy vegetables.

## 4. Conclusions

The paper proposes an automatic system for non-destructive growth measurement of potted leafy vegetables, based on Kinect v2 sensor. Multi-view point clouds of the measured plant were acquired with a turntable, then plant 3D reconstruction and growth parameter measurement were performed by processing these point clouds with a series of suitable algorithms, including segmentation, denoising, triangulation, etc. The system is capable of measuring plant height, total/projected leaf area and volume. In this paper, the principles of major algorithms were discussed and the key parameters were optimized to obtain satisfactory results.

Experiments were carried out by measuring 63 pots of lettuce in different growth stages. The result shows that, the height measured by our system has good linear relationship with reference measurements. More specifically, relative height shows better fitting goodness (*R*^2^ = 0.9914) than absolute height, while the soil method (*R*^2^ = 0.9855) has better accuracy than plant bottom method (*R*^2^ = 0.9457) for absolute height measurement. Besides, the measured projected leaf area also shows good linear relationship (*R*^2^ = 0.9510) with reference measurements. Whereas, the measured total leaf area (*R*^2^ = 0.9460) and volume (*R*^2^ = 0.9673) both follow power law distribution with reference data. In addition, the correlation study of Kinect measurements and biomass shows that, both the measured total leaf area and volume have general power law relationship with biomass. Kinect measurements have better correlations with fresh weight than dry weight, and volume can fit better with biomass than total leaf area. Among them, volume and fresh weight have the best fitting result (*R*^2^ = 0.9281). Therefore, it is feasible to estimate key growth parameters from Kinect measurements combining with the fitted formulas, with good accuracy. Furthermore, the system performance and the feasibility for future applications have been evaluated, showing good robustness and applicability.

As a comparison with present Kinect-based methods [28,29,30,31,32,33,37], the proposed system has advantages in achieving fine 3D reconstruction and accurate growth parameter measurement, but a major weakness of our method is the limited throughput due to the time-consuming problem. When compared to many other state of the art methods [4,5,7,8,9,10,15,19,20,23], the incomplete data (such as “holes”) and the lack of details can be a drawback for our reconstructed results, despite the low-cost advantage of our system. Therefore, the proposed system is more applicable to those applications which have limited budgets but low demands for precision and throughput.

The major contributions and advantages of our study are: The proposed system can obtain fine 3D reconstruction for leafy vegetables using a low-cost sensor; the whole workflow of scanning and data processing is automatic; key growth parameters can be measured accurately with a few properly selected or designed algorithms; and, the system has generally good robustness for different sizes, shapes, or species of green leafy vegetables.

However, the current study has a few limitations. First, the data processing speed can be influenced by the number of input points, which could be a time-consuming problem for large sized plant. Second, a stable lighting environment is required, since the segmentation of plant/non-plant is sensitive to color. Third, only truncated-cone-shaped pots can be used for our method, otherwise the in-pot parts cannot be segmented correctly. Fourth, each plant has to be put manually on the turntable, thereby limiting the application in automation scenarios.

Further works may include: Find a self-adaptive parameter selection method for the involved algorithms to make the system more flexible for different cases; Optimize algorithms or use other techniques (multithread, GPU, etc.) to speed up the time-consuming processes; Adapt the system to more species of leafy vegetables and test the robustness under different situations; Extra sensors can be introduced to obtain more complete information, such as measuring the plant from above with another camera; Make full use of the acquired colored 3D data, including point cloud and triangular mesh, to study and gain more growth and health information, such as stresses and diseases.

## Figures and Tables

**Table 1:** Segmentation results of plant and non-plant point clouds based on HSI color space.

Data Set	Plant Segmentation		Non-Plant Segmentation	
	TPR	TNR	TPR	TNR
Training Set	99.86%	99.46%	99.74%	99.89%
Test Set 1	99.91%	99.04%	99.19%	99.75%
Test Set 2	99.79%	99.63%	99.41%	99.96%
Test Set 3	99.85%	99.19%	99.89%	99.88%

**Table 2:** Descriptions and values of the key parameters used by our methods.

Parameter	Value	Description
*IterationTimes*	6	Maximum iteration times of MIE.
*MlsRadius*	0.01 (m)	Radius of MLS smoothing range.
*PointSpacing*	0.002 (m)	Minimum spacing of points for down-sampling.
*TriEdgeLength*	0.008 (m)	Maximum length of triangle edge for triangulation.
*BoundarySmoothR*	0.015 (m)	Radius of boundary smoothing range.
*MinSoilPoints*	10	Minimum allowable soil points number to get soil height.
*ProjAreaPrecision*	0.001 (m)	Spacing of matrix points for projected leaf area measurement.
*VolumePrecision*	0.0005 (m)	Spacing of matrix points for volume measurement.
*TetraEdgeLength*	0.008 (m)	Maximum length of tetrahedron edge for tetrahedralization.

**Table 3:** Details of the fitting results for reference—Kinect data and Kinect—biomass data.

Relation	Variables	Fitted Formula	*R*^2^	RMSE
Reference—Kinect	Relative Height	*y* = 1.002*x* − 0.7111	0.9914	0.2923 (cm)
	Absolute Height (Soil Method)	*y* = 1.021*x* − 0.5781	0.9855	0.3944 (cm)
	Absolute Height (Plant Bottom Method)	*y* = 0.9409*x* + 0.2327	0.9457	0.6957 (cm)
	Total Leaf Area	*y* = 44.17*x*^0.4321^ − 268.8	0.9460	72.43 (cm^2^)
	Projected Leaf Area	*y* = 0.788*x* + 1.252	0.9510	31.32 (cm^2^)
	Volume	*y* = 4.726*x*^0.5121^ − 3.911	0.9673	2.522 (cm^3^)
Kinect—Biomass	Total Leaf Area—Fresh Weight	*y* = 0.005783*x*^1.33^	0.8917	6.652 (g/cm^2^)
	Total Leaf Area—Dry Weight	*y* = 0.0001681*x*^1.436^	0.7781	0.6303 (g/cm^2^)
	Volume—Fresh Weight	*y* = 0.1949*x*^1.492^	0.9281	5.420 (g/cm^3^)
	Volume—Dry Weight	*y* = 0.006076*x*^1.669^	0.8402	0.5349 (g/cm^3^)

**Table 4:** Error of the measurements with linear relationships.

Measurement	MAE	MAPE
Relative Height	0.6550 (cm)	2.58%
Absolute Height (Soil Method)	0.4657 (cm)	5.46%
Absolute Height (Plant Bottom Method)	0.6070 (cm)	6.39%
Projected Leaf Area	85.49 (cm^2^)	21.59%

## Supplementary Materials

The following are available online at http://www.mdpi.com/1424-8220/18/3/806/s1. Supplementary data associated with this article have been provided. These data include the point clouds and meshes appeared in figures, the data sets used by scatter plots, and interactive MATLAB 3D scatter plots.
